# Supplementary figures and images for: LincRNA00612 inhibits apoptosis and inflammation in LPS-induced BEAS-2B cells via enhancing interaction between p-STAT3 and A2M promoter
Source: PeerJ. 2023 Mar 2;11:e14986. doi: 10.7717/peerj.14986 (PMC9985899; doi:10.7717/peerj.14986)

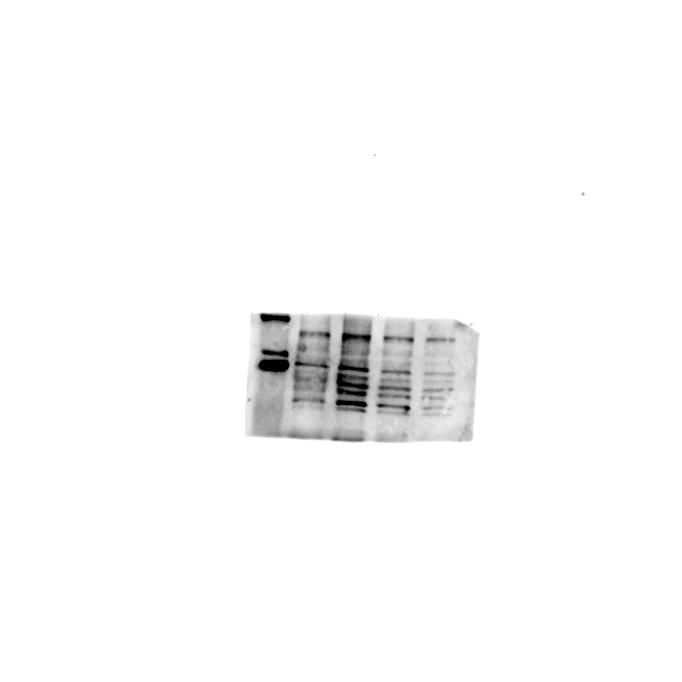

Supplement: Supplemental Information 5 [file peerj-11-14986-s005.zip › WB original images/A2M-3D.png]

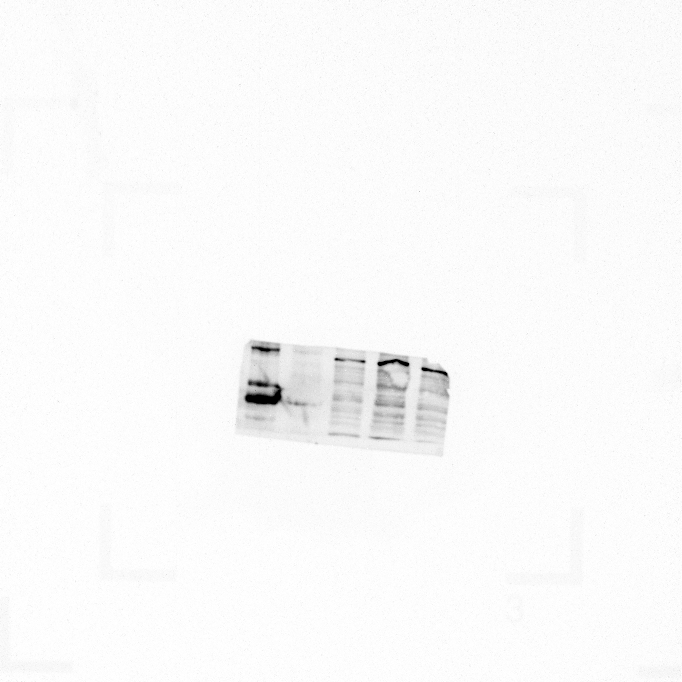

Supplement: Supplemental Information 5 [file peerj-11-14986-s005.zip › WB original images/A2M-3F.png]

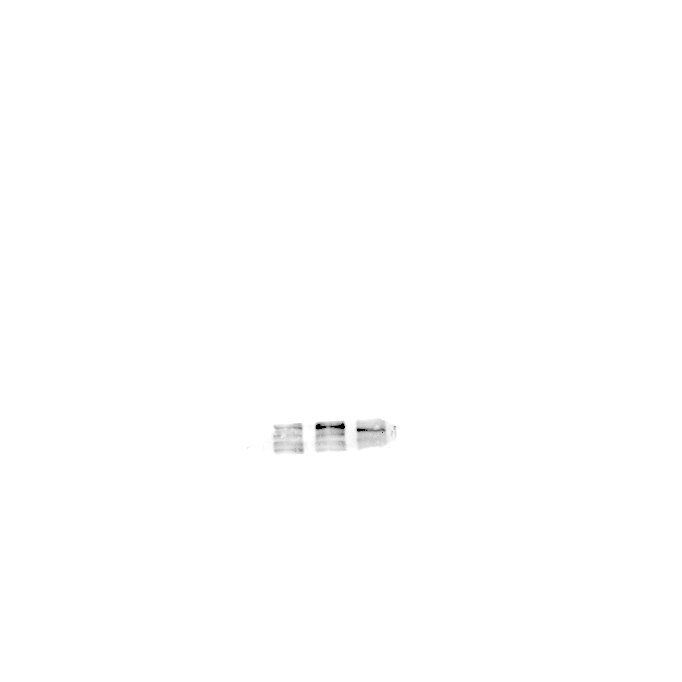

Supplement: Supplemental Information 5 [file peerj-11-14986-s005.zip › WB original images/A2M-6B.png]

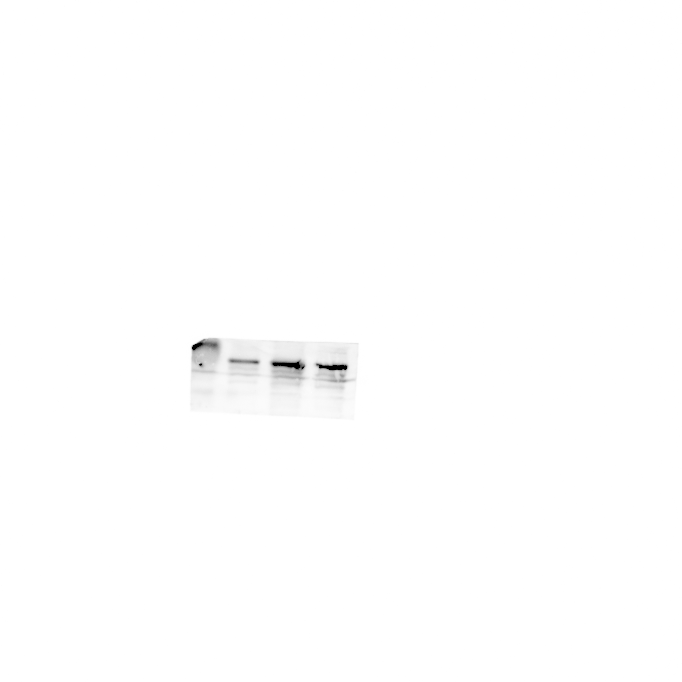

Supplement: Supplemental Information 5 [file peerj-11-14986-s005.zip › WB original images/cleaved c3-2D.png]

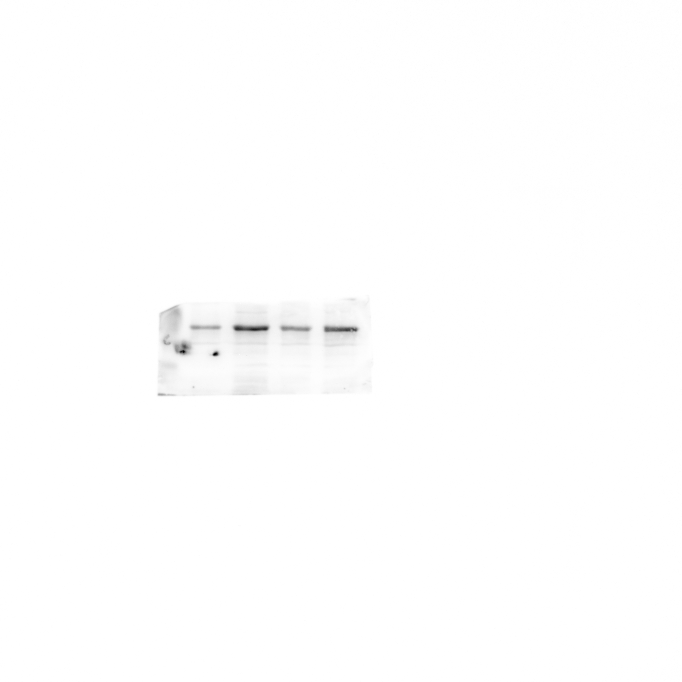

Supplement: Supplemental Information 5 [file peerj-11-14986-s005.zip › WB original images/cleaved c3-4C.png]

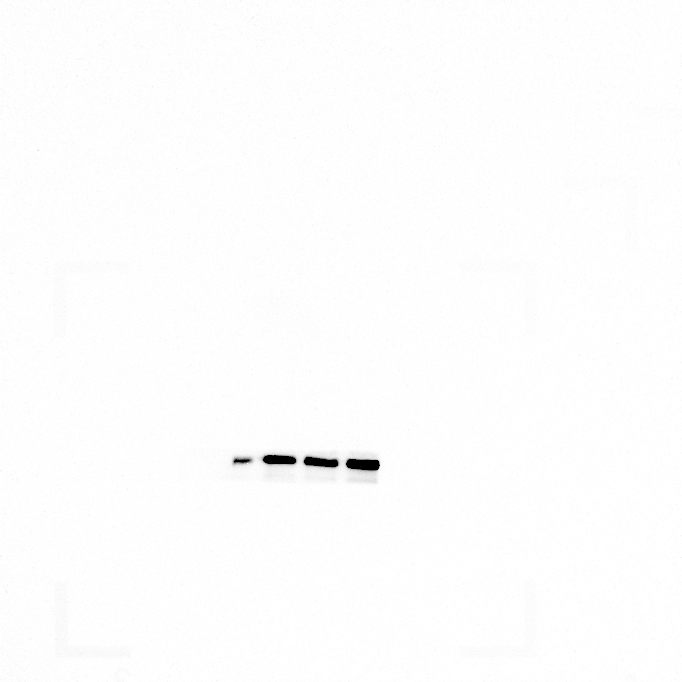

Supplement: Supplemental Information 5 [file peerj-11-14986-s005.zip › WB original images/gapdh-2D.png]

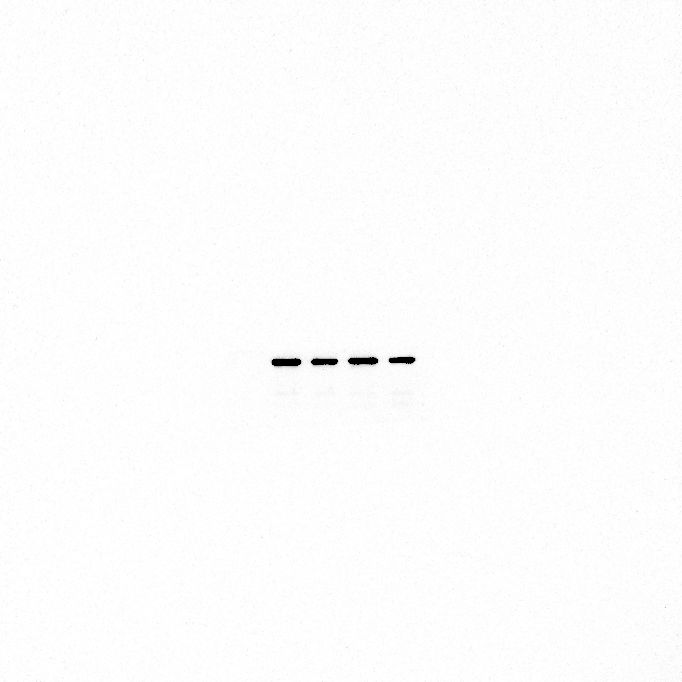

Supplement: Supplemental Information 5 [file peerj-11-14986-s005.zip › WB original images/gapdh-3D.png]

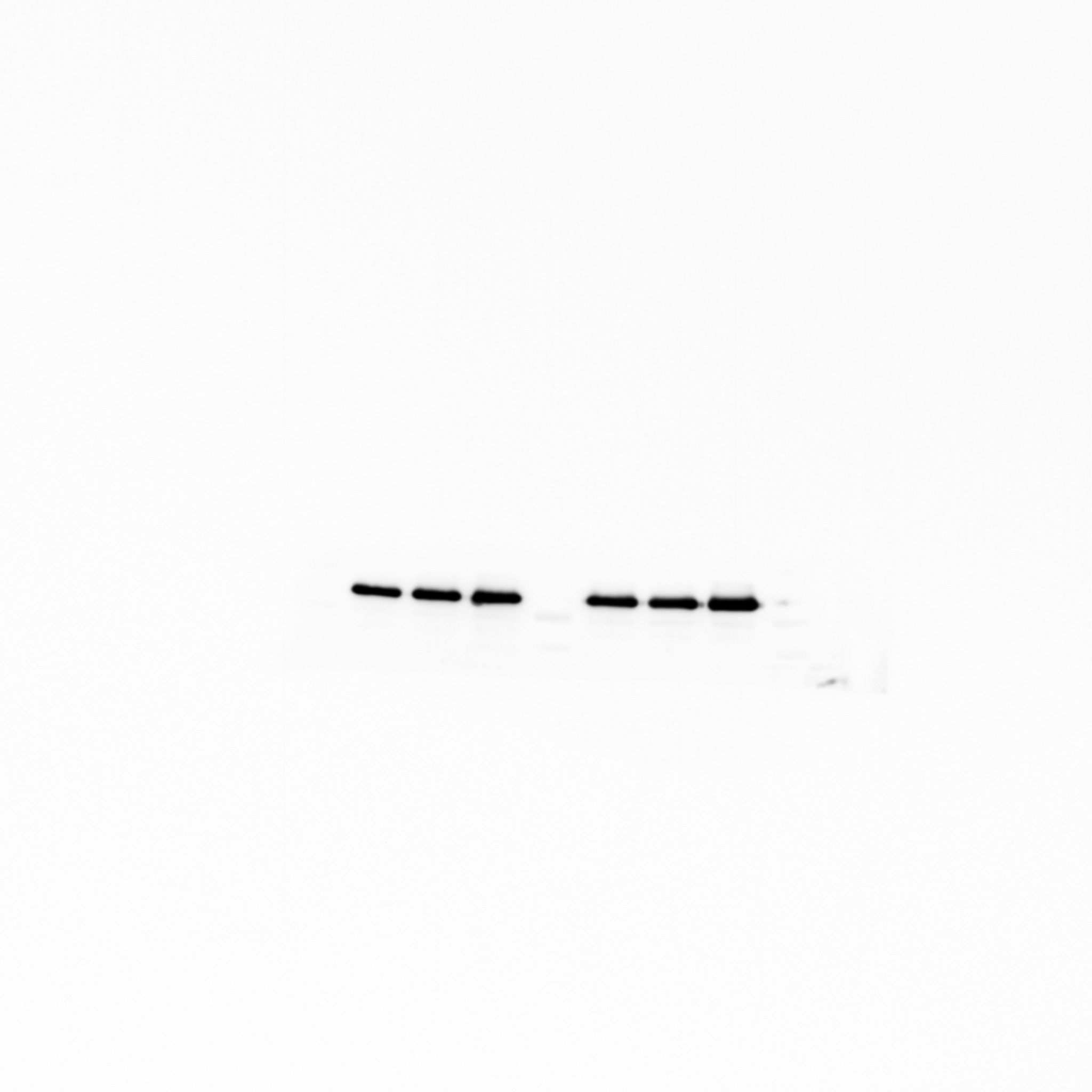

Supplement: Supplemental Information 5 [file peerj-11-14986-s005.zip › WB original images/gapdh-3F.png]

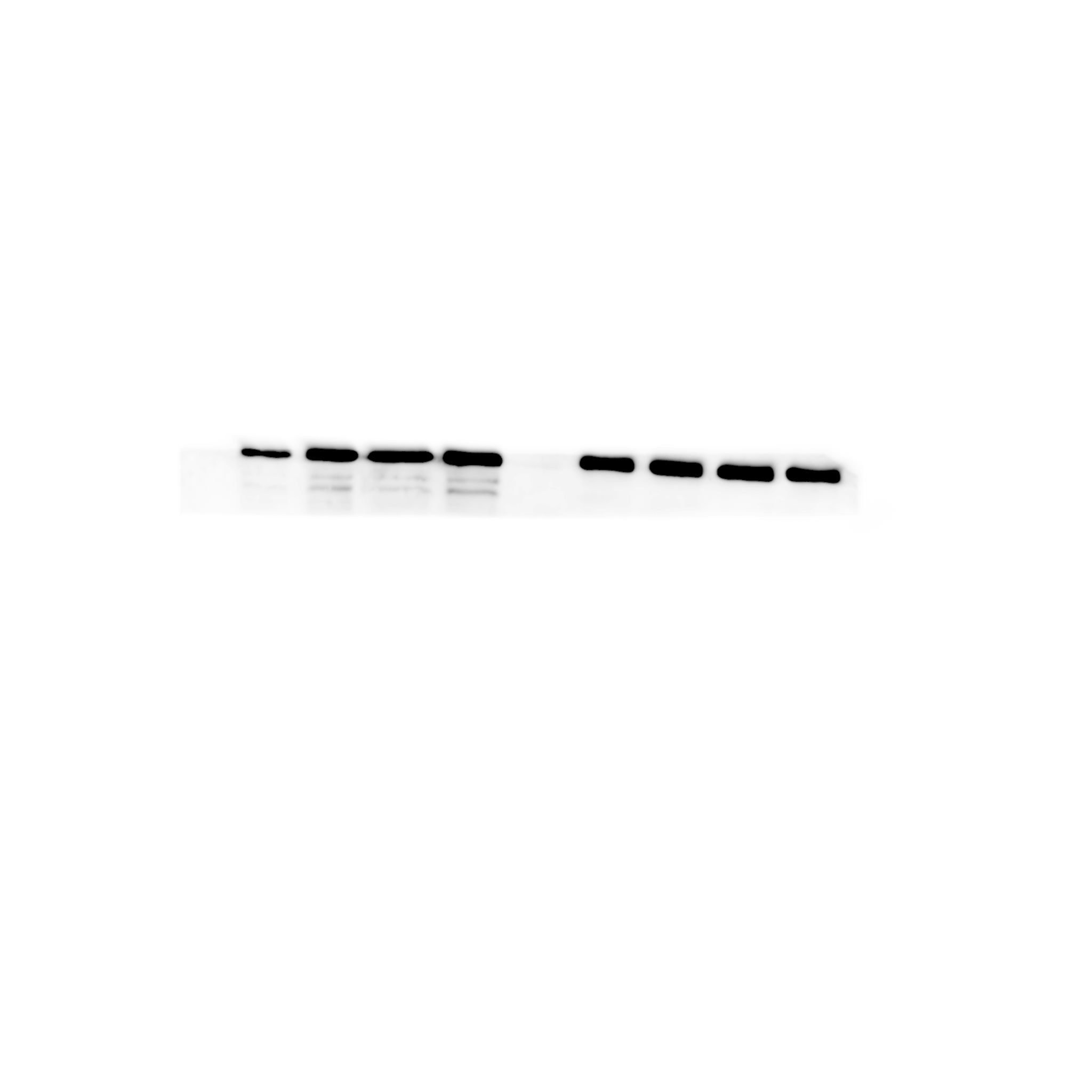

Supplement: Supplemental Information 5 [file peerj-11-14986-s005.zip › WB original images/gapdh-4C.png]

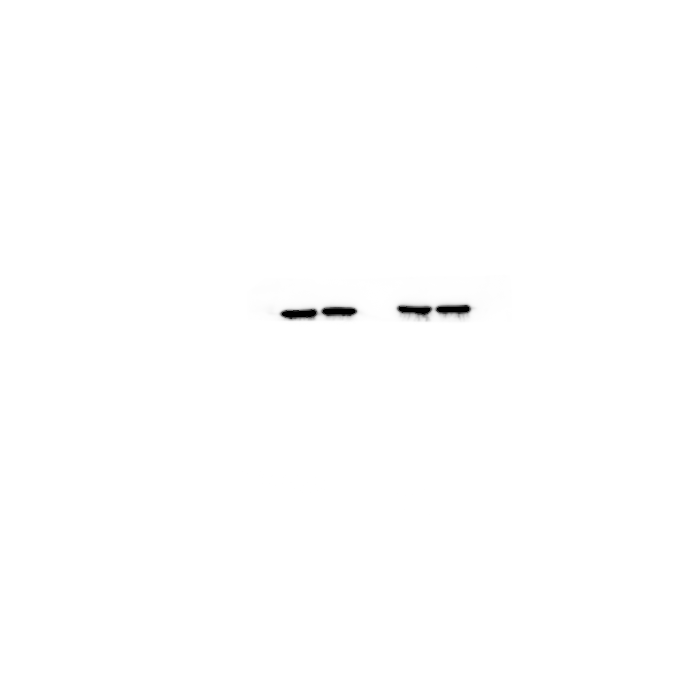

Supplement: Supplemental Information 5 [file peerj-11-14986-s005.zip › WB original images/gapdh-6B.png]

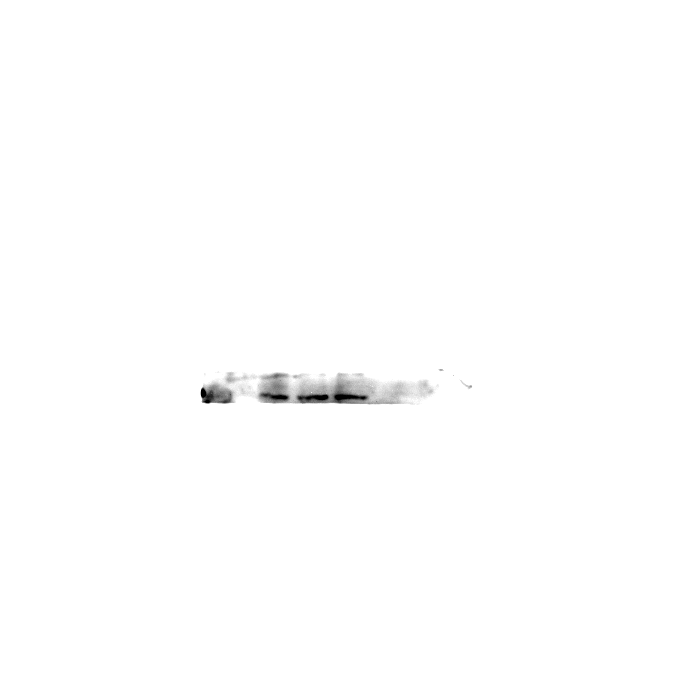

Supplement: Supplemental Information 5 [file peerj-11-14986-s005.zip › WB original images/pro c3-2D.png]

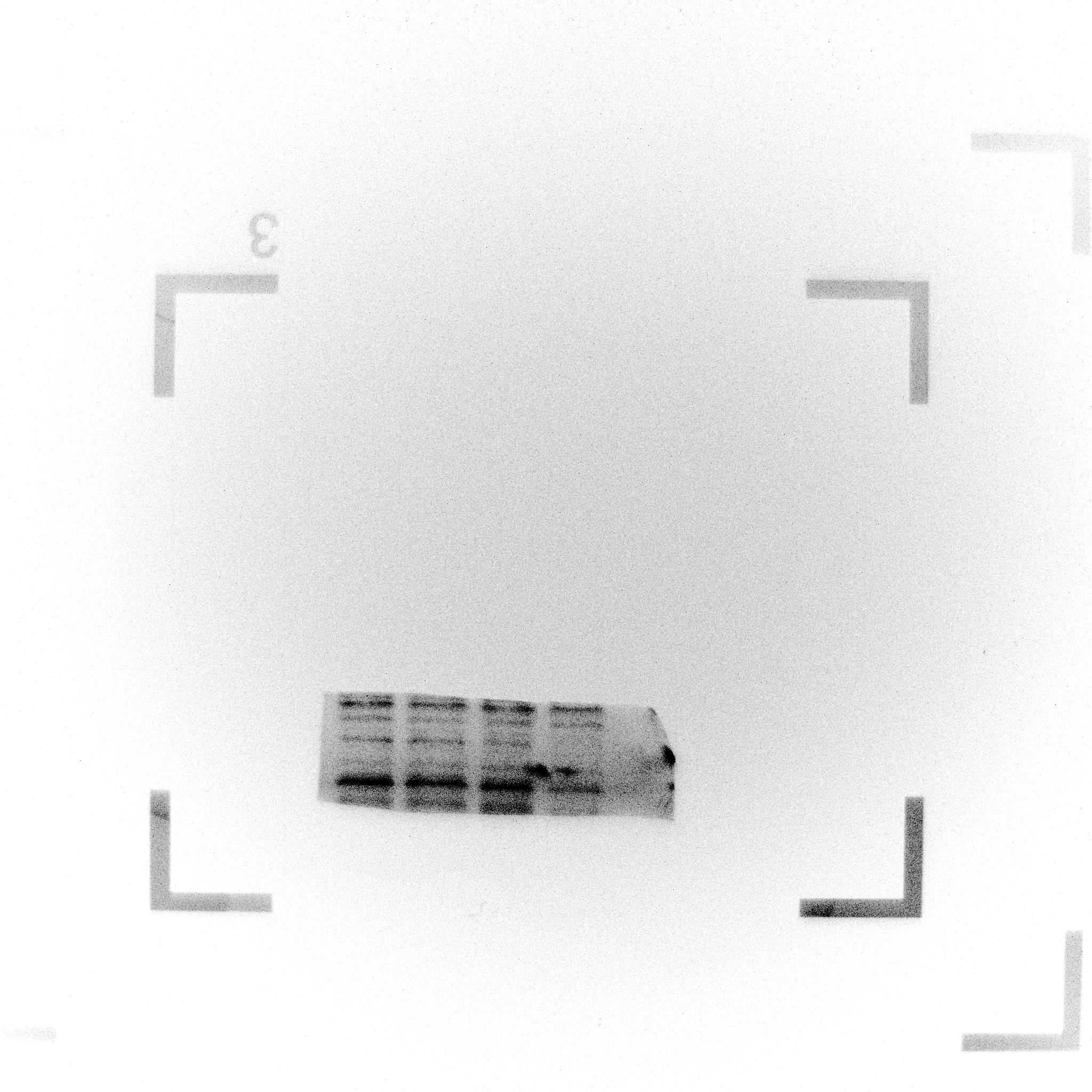

Supplement: Supplemental Information 5 [file peerj-11-14986-s005.zip › WB original images/pro c3-4C.png]

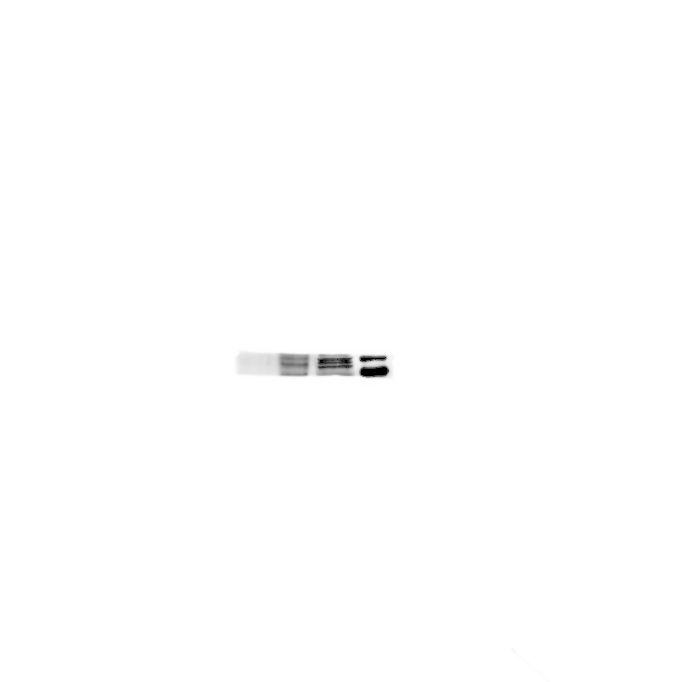

Supplement: Supplemental Information 5 [file peerj-11-14986-s005.zip › WB original images/pSTAT3-5B.png]

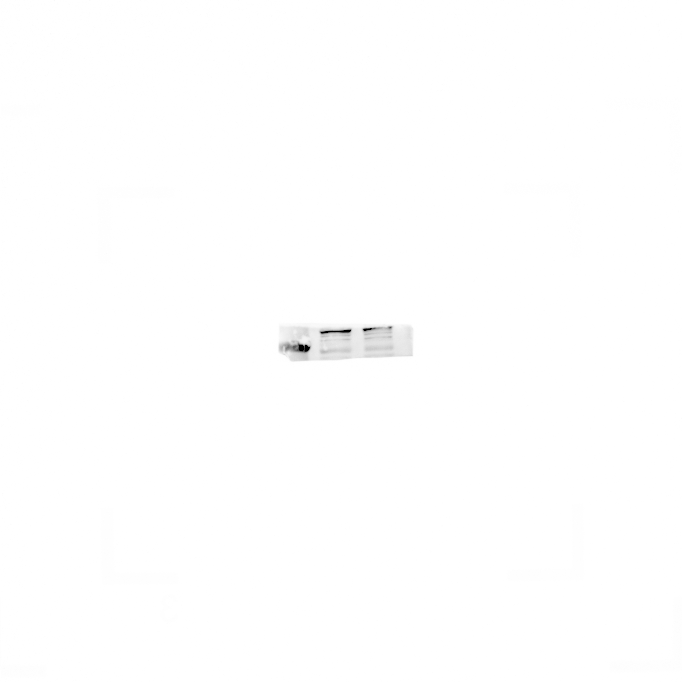

Supplement: Supplemental Information 5 [file peerj-11-14986-s005.zip › WB original images/pSTAT3-6B.png]

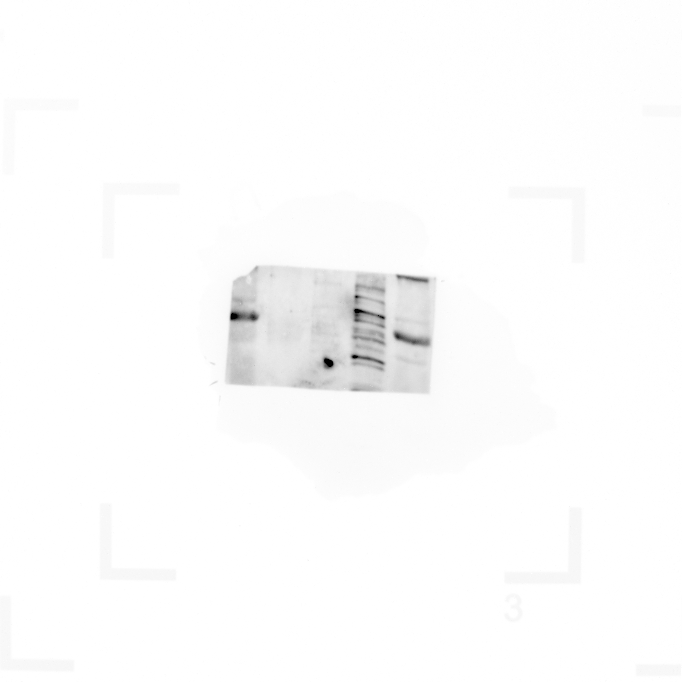

Supplement: Supplemental Information 5 [file peerj-11-14986-s005.zip › WB original images/tSTAT3-5B.png]

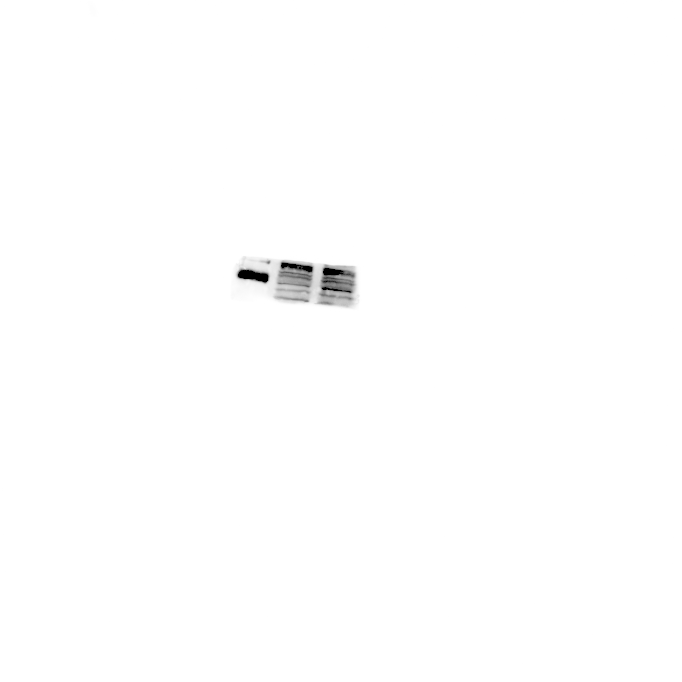

Supplement: Supplemental Information 5 [file peerj-11-14986-s005.zip › WB original images/tSTAT3-6B.png]
