# Supplementary material for: LincRNA00612 inhibits apoptosis and inflammation in LPS-induced BEAS-2B cells via enhancing interaction between p-STAT3 and A2M promoter
Source: PeerJ. 2023 Mar 2;11:e14986. doi: 10.7717/peerj.14986 (PMC9985899; doi:10.7717/peerj.14986)
Supplement: Supplemental Information 8 [file peerj-11-14986-s008.pdf]

Portal Home

*cat*RAPID fragments

[*cat*RAPID home - Documentation - Tutorial - Group page @ CRG]

Your submission results

Information about the JOB:

ID: 472808
User label: none
Protein sequence
Transcript sequence
Generated: 2022-06-29 03:48:57.889586

Interaction Z-score Profile (png)

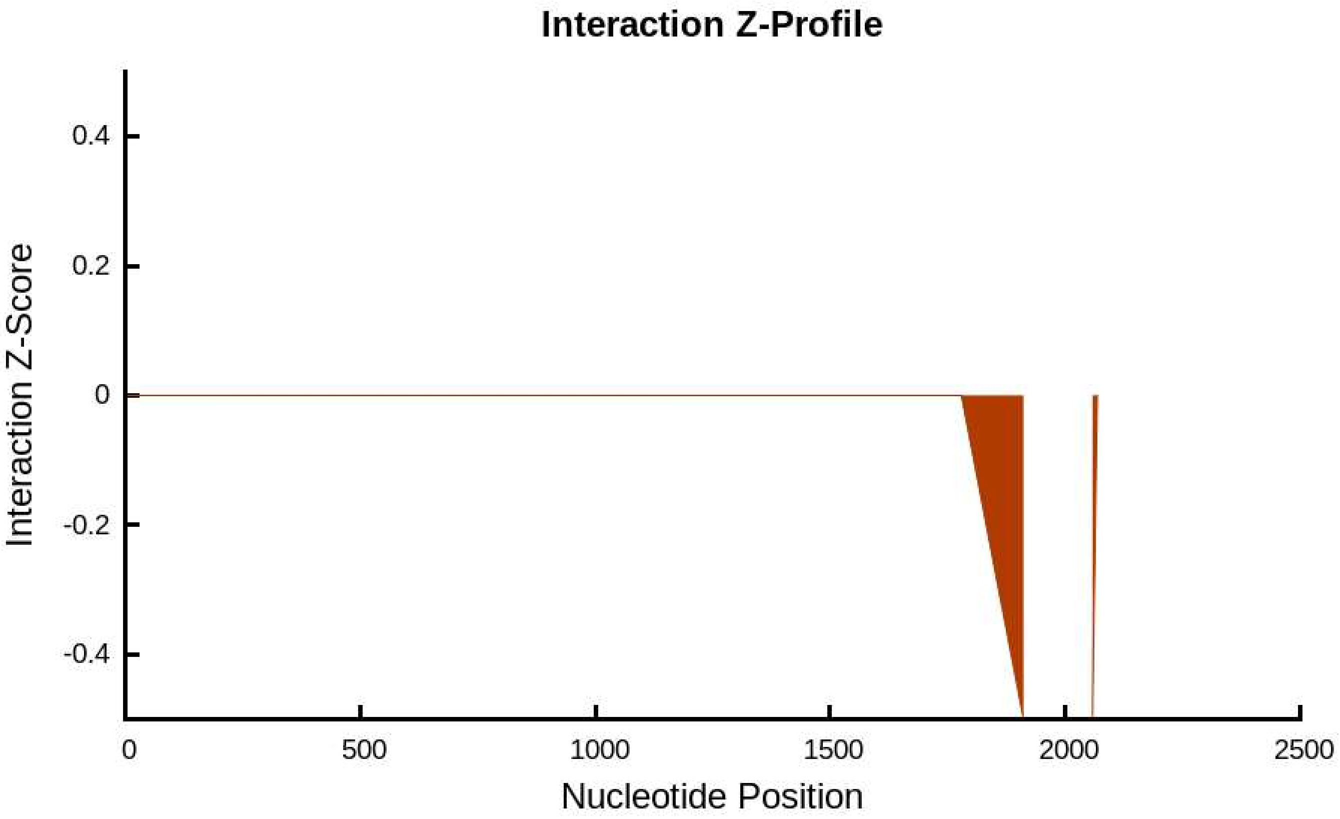

(**Download the text file of the results**):

#	Protein	RNA region	Interaction Propensity	Discriminative Power	Normalized Score
1	input_protei_1	51-169	11.30	33	2.05
2	input_protei_1	202-353	11.08	33	2.02
3	input_protei_1	141-245	10.77	32	1.97
4	input_protei_1	2056-2210	-1.90	14	0.06
5	input_protei_1	2108-2210	-3.68	14	-0.20
6	input_protei_1	2038-2211	-3.81	14	-0.22
7	input_protei_1	2082-2210	-4.01	14	-0.25
8	input_protei_1	2064-2211	-4.72	14	-0.36
9	input_protei_1	2091-2210	-4.86	14	-0.38
10	input_protei_1	2070-2202	-4.93	14	-0.39
11	input_protei_1	2076-2210	-5.03	14	-0.41
12	input_protei_1	2089-2210	-5.15	14	-0.42
13	input_protei_1	2101-2211	-6.01	14	-0.55
14	input_protei_1	2023-2211	-8.30	14	-0.90
15	input_protei_1	2029-2216	-9.55	10	-1.09
16	input_protei_1	2033-2216	-10.75	10	-1.27

[illegible]	[illegible]	[illegible]	[illegible]	[illegible]	[illegible]

© 2012 - Gene Function and Evolution - Center for Genomic Regulation (CRG)
